# Surgical management of plexiform neurofibromas: An imaging-based perioperative framework

**DOI:** 10.1016/j.jpra.2026.08.006

**Published:** 2026-08-19

**Authors:** Kevin Ghajar, Adham Elsherbini, Emily Volfson, Rebecca Yakubov, Heather L. Baltzer, Suganth Suppiah, Kevin J. Zuo

**Affiliations:** a Temerty Faculty of Medicine, University of Toronto, Toronto, Canada; b Faculty of Medicine, University of Ottawa, Ottawa, Canada; c Division of Plastic, Reconstructive and Aesthetic Surgery, Department of Surgery, University of Toronto, Toronto, Canada; d Division of Plastic Surgery, University Health Network, Toronto, Canada; e Division of Neurosurgery, Department of Surgery, University of Toronto, Toronto, Canada

**Keywords:** Flow-void sign, Malignant peripheral nerve sheath tumor, Neurofibromatosis type 1, Plexiform neurofibroma, Preoperative embolization, Reconstructive planning, Surgical management

## Abstract

Plexiform neurofibromas (PNs) are complex, infiltrative peripheral nerve sheath tumors affecting up to half of individuals with neurofibromatosis type 1 (NF1). Their diffuse, multicompartment growth, potential for disfigurement and neurologic compromise, and elevated risk of malignant peripheral nerve sheath tumor make management uniquely challenging. Optimal care hinges on accurate imaging, multidisciplinary planning, and strategic selection between complete resection and function-preserving debulking. MRI is central to perioperative assessment, with whole-body MRI supporting baseline mapping and regional MRI providing high-resolution definition for operative planning. Angiographic studies assist in identifying candidates for preoperative embolization, while FDG-PET improves detection of distinct nodular lesions and atypical neurofibromas with higher malignant potential. Preoperative embolization may reduce intraoperative hemorrhage in highly vascular PNs. Surgical indications include progressive neurologic deficits, airway or organ compromise, refractory pain, and severe disfigurement. Complete resection offers maximal oncologic control but risks permanent deficits; subtotal or debulking procedures prioritize function preservation while accepting recurrence risk. Adjuncts such as fluorescein-guided resection, intraoperative neurophysiological monitoring, and 3-dimensional planning enhance precision and safety. Reconstruction requires site-specific strategies for head and neck, truncal, and extremity lesions. Postoperatively, MRI-based surveillance is critical, particularly following subtotal resection or in high-risk anatomical locations. MEK inhibitors, including selumetinib, provide effective non-surgical therapy for progressive or unresectable PNs. Management of PNs demands individualized, multidisciplinary care. An imaging-driven approach, selective use of embolization, and incorporation of intraoperative adjuncts can optimize tumor control and functional outcomes. Prospective multicenter studies are needed to standardize imaging protocols, surgical selection criteria, and long-term outcome reporting.

## Introduction

Neurofibromatosis type I (NF1) is an autosomal dominant tumor predisposition syndrome resulting from pathogenic mutations in the *NF1* tumor suppressor gene, leading to dysregulated Ras/MAPK signalling and formation of peripheral nerve sheath tumors.^1^ While cutaneous neurofibromas are nearly universal in NF1, plexiform neurofibromas (PNs) represent a biologically and clinically distinct subtype that affects 30–50% of patients.^2^, ^3^, ^4^ PNs involve several peripheral nerve branches and demonstrate an infiltrative, poorly circumscribed growth pattern that can lead to displacement or extension into skin, bones, and internal organs.^4^, ^5^, ^6^, ^7^, ^8^ Unlike cutaneous neurofibromas, PNs have a much higher risk of malignant transformation.^1^, ^2^, ^3^, ^4^, ^5^, ^6^, ^7^, ^8^

Clinically, PNs typically present in childhood and may arise anywhere in the body, although they are most commonly encountered in the head and neck region, pelvis, and lower extremities.^1^, ^2^, ^3^, ^4^ Among these lesions, head and neck PNs warrant particular attention because their proximity to the airway, orbit, cranial nerves, and major vascular structures makes operative planning especially complex and underscores the value of an imaging-based perioperative approach. Morbidity from PNs largely reflects the tumour’s effect on adjacent structures and may include pain, cosmetic disfigurement, focal neurologic deficits, and airway compromise, all of which may prompt surgical intervention^1^^,^^4^, ^5^, ^6^, ^7^ Surgical resection, however, may have significant risks, including nerve injury, hemorrhage, impact on adjacent organs, and unsatisfactory aesthetic results.^4^, ^5^, ^6^, ^7^, ^8^ Currently, there is no established classification for surgical candidacy.

This review provides an overview of the perioperative management of PNs, highlighting the use of magnetic resonance imaging (MRI) for preoperative embolization and focusing on specific surgical resection and reconstructive techniques.

## Methods

A comprehensive literature search was conducted from database inception through May 2026. The search strategy incorporated combinations of medical subject headings and free-text terms including “plexiform neurofibroma”, “neurofibromatosis type 1″, “peripheral nerve sheath tumor”, “surgical management”, “resection”, “debulking”, “preoperative imaging”, “magnetic resonance imaging”, “angiography”, “preoperative embolization”, “reconstruction”, and “malignant peripheral nerve sheath tumor” and their synonyms.

Eligible studies included primary research articles, systematic reviews, and meta-analyses published in English that investigated the imaging evaluation, perioperative planning, surgical management, and postoperative outcomes of PNs. Studies addressing malignant transformation, including atypical neurofibromas and malignant peripheral nerve sheath tumors, were also included where relevant to surgical decision-making.

### Preoperative imaging for surgical planning

Imaging is central to perioperative decision-making for PNs and should be selected to answer the specific clinical question. Whole-body MRI (WB-MRI) is most useful at baseline or when disease is widespread (e.g., screening for multiple neurofibromas or mapping very large lesions that cross anatomical compartments) .^7^ In contrast, regional MRI with an optimized field-of-view is chosen for high-resolution preoperative assessment of a single PN because it better defines the lesion’s margins and relationships to adjacent neurovascular and musculoskeletal structures.^7^ When vascular anatomy will affect operative strategy, MR angiography or computed tomography (CT) angiography provides complementary mapping of arterial feeders and helps identify candidates for preoperative embolization.^7^ Fluorodeoxyglucose-Positron Emission Tomography (FDG-PET) (alone or PET/CT or PET/MRI) adds metabolic information and is useful for differentiating benign from malignant change, offering high sensitivity and moderate–high specificity when interpreted alongside MRI and clinical features (e.g., new pain or rapid growth)^9^, ^10^, ^11^

MRI remains the workhorse for surgical planning because of its superior soft-tissue contrast.^7^^,^^12^^,^^13^ Surgical protocols typically include T1- and T2-weighted sequences with fat suppression (STIR or fat-sat T2) and post-contrast T1 to reveal infiltrative components, internal nodularity, and abnormal enhancement that impact resectability.^7^^,^^13^ MRI also highlights imaging features that raise concern for malignant transformation: heterogeneous T1/T2 signal, necrosis or hemorrhage, irregular or peripheral contrast enhancement, perilesional edema, and rapid interval enlargement which should prompt expedited multidisciplinary assessment and consideration of FDG-PET or tissue sampling.^14^^,^^15^

Beyond standard sequences, volumetric MRI enables objective 3D measurement of tumor volume and growth kinetics.^7^^,^^16^ Serial volumetrics increase sensitivity for clinically meaningful change and help time intervention or evaluate treatment response.^7^^,^^16^ For anatomically complex or head and neck PNs, 3D reconstruction from MRI/CT provides patient-specific models of tumor, entrapped nerves, vessels, and bone deformity that support preoperative simulation, osteotomy planning, and fabrication of cutting guides to improve resection precision.^12^

Despite these advances, imaging protocols remain heterogeneous across centres. Until standardized criteria are available, imaging should be individualized according to lesion size, location, growth behavior, symptoms, prior interventions, and the planned surgical approach.^17^, ^18^, ^19^

### Malignancy evaluation: distinct nodular lesions and atypical neurofibromas

Before initiating any definitive PN-directed therapy, clinicians must exclude malignant transformation.^7^ A change in growth kinetics, new or worsening pain, neurologic decline, or rapid change in tumor consistency should prompt urgent reassessment for a malignant peripheral nerve sheath tumor (MPNST) because management and prognosis differ substantially.^20^^,^^21^ When tissue diagnosis is required, biopsy should be planned to preserve future resectability and to minimize additional neurologic risk (image-guided core or limited open biopsy through a corridor that can be included in a subsequent definitive resection if needed)^20^, ^21^, ^22^ If MPNST is confirmed, treatment is oncologic, wide excision with negative margins and multidisciplinary sarcoma care combined with adjuvant radiotherapy or chemotherapy.^21^^,^^22^

The lifetime risk of MPNST in NF1 is substantial, with most cases diagnosed between ages 20 and 40, and the majority arising from preexisting PNs, particularly distinct nodular lesions (DNLs) and atypical neurofibromas (ANFs) .^23^^,^^24^ DNLs are radiologic entities: discrete, well-circumscribed nodules arising within or adjacent to a PN. They often differ biologically and behaviorally from diffuse PNs, are frequently FDG-PET–avid, more commonly symptomatic (pain, focal neurologic signs), and may show age-independent growth dynamics.^24^^,^^25^ ANFs are defined histopathologically by increased cellularity, nuclear atypia, and frequent loss of CDKN2A/B; molecular profiling places them between benign neurofibromas and frank MPNST, with early oncogenic and immune alterations that presage malignant transformation.^26^^,^^27^

Because DNLs and ANFs carry higher transformation risk than conventional PNs, they warrant intensified, risk-adapted surveillance. Regional MRI at shorter intervals and adjunctive FDG-PET or PET/MRI should be considered when clinical or imaging red flags are present, including rapid interval growth, new pain, neurologic change, suspicious nodular transformation, or atypical enhancement.^23^ Azizi et al. showed that [¹⁸F] FDG-PET may help identify potentially malignant PNs even in asymptomatic children and adolescents with NF1.^28^ However, because FDG uptake can overlap between benign and malignant lesions, PET findings should be interpreted alongside symptoms, MRI morphology, interval growth, and multidisciplinary assessment rather than used as a standalone screening test. Emerging approaches such as fibroblast activation protein (FAP)-targeted PET may improve specificity in equivocal FDG-PET-avid lesions, but these strategies remain investigational and require further validation.^29^

### Preoperative embolization

**Fig. 1:**
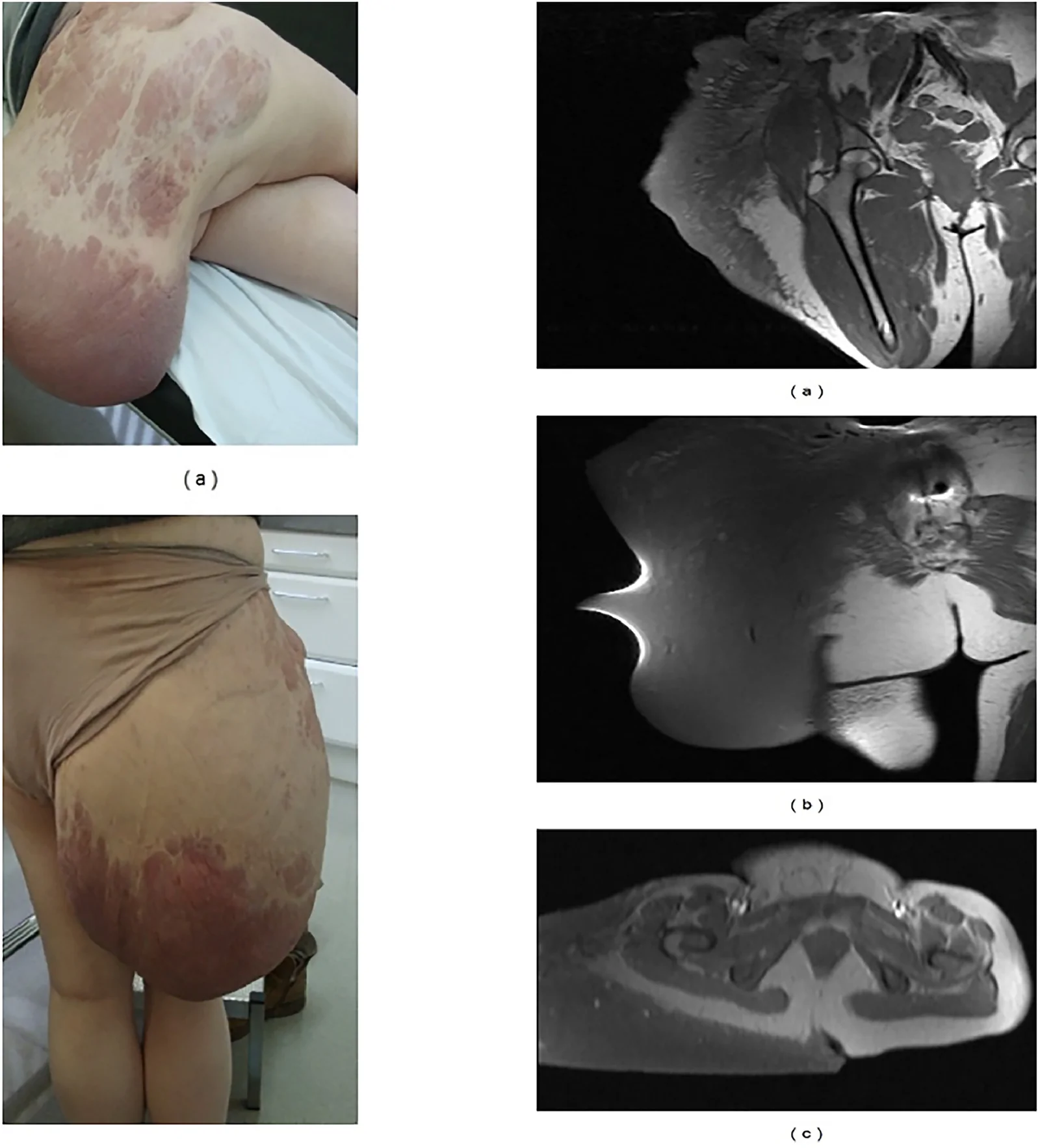
Preoperative images of a giant PN in the right buttock and lumbar region with associated MRI images. Note in the MRI images that there is a flow void, which is lack of image definition in the area of the PN due to the high flow vascularity within the tumour. Reproduced with permission from Vélez et al. 2013, Case Reports in Neurological Medicine, licensed under CC BY 4.0 (https://creativecommons.org/licenses/by/4.0/).

MRI can identify PNs with high vascularity, most notably via the “flow-void” sign on T2-weighted or contrast-enhanced sequences which predicts a heightened risk of intraoperative hemorrhage and postoperative hematoma (Fig. 1) .^30^ In patients with large PNs demonstrating a positive flow-void sign, preoperative embolization may be useful to reduce tumor vascularity and intraoperative hemorrhage and facilitate safer, more complete resection.^30^^,^^31^

Embolization is typically performed 1–2 days before surgery and involves angiography to occlude feeding blood vessels with polyvinyl alcohol particles or liquid embolics (e.g., Onyx) depending on vessel caliber and flow dynamics.^31^ Only lesions with clear evidence of a major arterial supply stand to benefit from this technique.

Vélez et al. (2013) and Tovo Filho et al. (2020) describe similar pragmatic approaches to giant, highly vascular PNs: detailed angiographic vascular mapping followed by targeted preoperative embolization then planned surgical resection.^32^^,^^33^ Both reports show that embolization can achieve near-complete devascularization, improve resectability, and reduce intraoperative hemorrhage. However, embolization does not always eliminate transfusion need and may cause trade-offs such as skin ischemia, necrosis, and wound complications that require reconstruction.

Together, these reports emphasize that preoperative embolization is a useful adjunct for select, highly vascular PNs, but additional prospective data and standardized protocols are needed to select appropriate candidates and plan reconstruction.

### Surgical intervention

Surgery remains the principal treatment for PNs when intervention is indicated. Acute indications include actual or impending neurological compromise and impingement of vital structures (e.g., airway) while relative indications are those aimed at improving quality of life, such as refractory pain or severe disfigurement. Decisions to operate are individualized by weighing tumor location, growth behaviour, patient age, and the anticipated balance between symptomatic benefit and risk of treatment-related neurologic morbidity.^4^, ^5^, ^6^, ^7^, ^8^ Prophylactic or cosmetic surgery may also be considered; however, its effectiveness remains uncertain.

Because PNs arise from multiple fascicles and infiltrate surrounding soft tissues, clear dissection planes rarely exist. Consequently, surgical goals are framed in terms of extent of resection, namely complete versus subtotal or debulking.

#### Complete resection

Complete resection aims to remove the entire tumor while preserving neurologic function whenever possible; however, the operative strategy differs based on lesion type. For benign PNs, the tumor may be separable from the parent nerve, with sacrifice limited to the feeding fascicle rather than the entire nerve.^34^ This distinction is important because segmental nerve resection and nerve reconstruction are not required for all PNs. In contrast, when MPNSTs are present or strongly suspected, oncologic resection with negative margins becomes the priority and may require segmental parent nerve resection.^7^ Although this approach offers the greatest likelihood of tumor clearance, it sacrifices nerve function, may or may not be reconstructible, and therefore carries a risk of permanent neurologic deficit.^6^^,^^7^^,^^35^

These distinctions further emphasize the importance of preoperative high-resolution ultrasound and MRI in characterizing lesion morphology, defining the relationship between the tumor and parent nerve, and estimating the expected neurologic deficit if resection is pursued.

In retrospective series, complete resection has produced symptomatic relief but also measurable rates of new neurologic deficits. For example, in a series of 24 deep-seated nodular PNs, eight required complete removal and four patients developed new deficits (two persistent) post-surgery.^6^ The decision to pursue complete resection should therefore be reserved for cases where oncologic necessity or life-/organ-threatening compromise outweighs the functional cost.

#### Subtotal / debulking resection

When complete excision would cause unacceptable neurologic or cosmetic morbidity or significant hemorrhage, subtotal or function-sparing debulking is commonly preferred. In this strategy, surgeons focus on removing mass-effecting and symptomatic portions of the lesion to relieve pain, compression, or disfigurement, while deliberately leaving residual tumour in situ, accepting a higher likelihood of residual disease and future regrowth.^20^

#### Head and neck plexiform neurofibromas: special surgical considerations

Head and neck PNs represent a clinically important subgroup in which the value of an imaging-based perioperative framework is particularly evident. In a landmark surgical cohort of 121 patients with NF1-associated PNs undergoing 302 procedures over 20 years, head, face, and neck tumours were the most common anatomical locations (42.9%), followed by the extremities (28.0%) and the trunk, spine, and internal organs (29.1%). Younger age and head and neck location were independent predictors of earlier tumour progression following resection.^36^ Consistent with this, a pediatric-specific nationwide survey identified the face (39%), neck (27%), and head (24%) as the most frequent tumor locations in children with NF1, underscoring the disproportionate burden of this region.^37^ These lesions can cause pain, cosmetic disfigurement, airway or swallowing compromise, and reduced quality of life.^38^ When cranial nerve ganglia are involved, the risk of malignant transformation may also increase.^39^ Accordingly, early intervention may be warranted in selected pediatric patients with large or progressive head and neck PNs to prevent tracheostomy dependence, dysphagia, and progressive facial disfigurement.^40^

However, surgical management is challenging because these tumors are often deeply infiltrative, hypervascular, and closely associated with cranial nerves, major vessels, and the orbit. Preoperative MRI is therefore central to operative planning, as it allows surgeons to classify lesions as superficial, displacing, or invasive, thereby guiding the feasibility of complete versus subtotal resection, anticipating reconstructive requirements, and identifying patients who may benefit from adjunctive measures such as angiography, embolization, intraoperative neurophysiological monitoring (IONM), staged resection, or planned reconstruction.

The extent of resection is determined by lesion depth, morphology, and functional risk. Superficial lesions may be amenable to near-complete excision with durable cosmetic improvement, whereas invasive tumors traversing multiple tissue planes usually require subtotal or staged debulking to avoid unacceptable neurologic, functional, or aesthetic morbidity.^13^ Orbitotemporal disease requires particular caution; anterior orbital lesions may respond well to debulking, whereas retrobulbar extension often involves the levator muscle, ocular nerves, and orbital bone, limiting the extent of safe resection.^41^ Surgery may also be required to relieve airway obstruction or dysphagia, although residual disease and regrowth remain important concerns, particularly in young patients with head and neck lesions.^42^

Because complete excision is often not feasible, staged resection combined with tailored reconstruction has emerged as a pragmatic strategy that prioritizes functional preservation and contour improvement over radical excision.^43^ In a recent series of large facial PNs, patients underwent an average of four operations; near-total excision (>90%) was achieved in 4 patients (21%), while most underwent subtotal or planned staged partial excision.^43^ Lesion morphology further influences operative strategy: nodular PNs may be treated with enucleation when a safe plane exists, whereas en bloc resection with nerve ligation is reserved for cases in which the parent nerve is involved.^6^

Hemorrhage control is another major consideration because these tumors are frequently hypervascular. Reported strategies include tumescent infiltration, bipolar coagulation scissors, systematic ligation of venous confluents, fibrin sealant spray, and circumferential parallel ligation for very large tumors.^44^^,^^45^ These techniques have been associated with low transfusion requirements in select patients.^44^^,^^45^ Chen et al. reported two patients with massive NF1-associated head and neck PNs undergoing total resection (tumours weighing 6.5 kg and 1.5 kg), in which intraoperative haemostasis was achieved using ultrasonic shears in advanced haemostasis mode combined with systematic vessel ligation, limiting blood loss during tumour resection to 1500 mL and 600 mL, respectively, with only one patient requiring transfusion.^46^ The resulting composite defects were then reconstructed using the extended vertical lower trapezius island myocutaneous flap (eTMF) pedicled on the transverse cervical vessels. Both flaps were harvested successfully with no serious complications, ECOG performance status recovered to grade 0 by week 8, and swallowing, chewing, and speech returned to preoperative levels with quality of life improving by 40% at 6 months, without significant donor-site morbidity.^46^

#### Adjunctive intraoperative techniques

Adjunctive intraoperative techniques may improve resection precision and reduce neurologic morbidity in complex PN surgery. Multimodal IONM, including motor evoked potentials (MEP), electromyography (EMG), somatosensory evoked potentials (SEP), and visual evoked potentials (VEP), has been used safely in head and neck NF1 tumor surgery, enabling real-time detection of nerve irritation or injury and helping distinguish functional from nonfunctional.^47^^,^^48^ Fluorescein-guided resection may further aid tumor visualization by highlighting tumor tissue under a specialized microscope filter, and comparative series have reported higher gross total resection rates with fluorescein guidance without increased complications.^49^

### Reconstruction & cosmetic outcomes

#### Head and neck plexiform neurofibromas

Reconstruction after head and neck PN resection should be individualized according to lesion size, location, vascularity, and anticipated defect. In unilateral hemifacial PNs, facial aesthetic unit remodeling can guide contour restoration by using the unaffected hemiface as a template for planned volumetric restoration.^45^ Rather than relying on conventional debulking, this strategy employs preoperative aesthetic-unit mapping, controlled translesional resection, and adjunctive hemostatic measures to improve function and cosmesis while minimizing bleeding.

For large facial PNs, staged excision with immediate reconstruction is a pragmatic strategy because complete excision is limited by infiltrative growth, hypervascularity, and extensive soft-tissue hypertrophy. Local or regional flaps are commonly used for staged contour restoration and have been associated with reliable flap survival, improved facial symmetry, high patient-reported aesthetic satisfaction, and functional recovery.^43^^,^^50^

For larger composite defects, regional flaps or free tissue transfer may be required when local tissue is insufficient. Options such as the extended vertical lower trapezius island myocutaneous flap, anterolateral thigh flap, and latissimus dorsi myocutaneous flap can provide durable soft-tissue coverage and support more aggressive resection in selected extensive or recurrent cases, although substantial blood loss remains a major perioperative concern.^46^^,^^51^

Tissue expansion is another option when optimal color, texture, and thickness match is desired, particularly for visible head and neck defects.^52^ However, it requires staged surgery, prolonged planning, and careful hemostatic management. Long-term durability may depend on donor-site selection, as NF1-associated skin hyperextensibility may compromise contour stability in gravity-dependent regions.^53^

Overall, reconstruction in head and neck PN surgery should prioritize functional preservation, facial symmetry, durable soft-tissue coverage, and patient-centered aesthetic outcomes.

#### Trunk

Truncal PNs often give rise to secondary musculoskeletal deformities, necessitating coordinated surgical planning. In one case report, a 12-year-old boy developed pronounced pectus excavatum and significant cosmetic distortion secondary to a large anterior chest wall PN.^54^ The surgeon first corrected the sternal depression using a minimally invasive Nuss technique, followed by a complete resection of the 21 × 17 × 5 cm lesion. Because the tumour lay superficially, sparing muscle and rib structures, the resection specimen was removed intact, and the skin defect was closed primarily without tension. Both the patient and his family reported high satisfaction with the restored thoracic contour, and a four-year follow-up demonstrated maintained chest wall integrity and no recurrence. In contrast, PNs of the lumbosacral region present a different set of hurdles, specifically massive tumour bulk and hypervascularity. A case report of a 22-year-old woman with a giant lumbosacral PN accounting for over 20% of her body weight illustrates the necessity of a multidisciplinary strategy.^32^ Preoperative arterial embolization in two stages dramatically reduced tumour blood flow. During resection, an endoscopic linear stapler was used to seal the feeding vessels, thereby minimizing intraoperative hemorrhage and transfusion requirements. The resultant soft-tissue defect was reconstructed using a local rotational flap to facilitate tension-free closure. The patient regained full mobility postoperatively, and two-year imaging confirmed no tumour regrowth.

#### Extremities

**Fig. 2:**
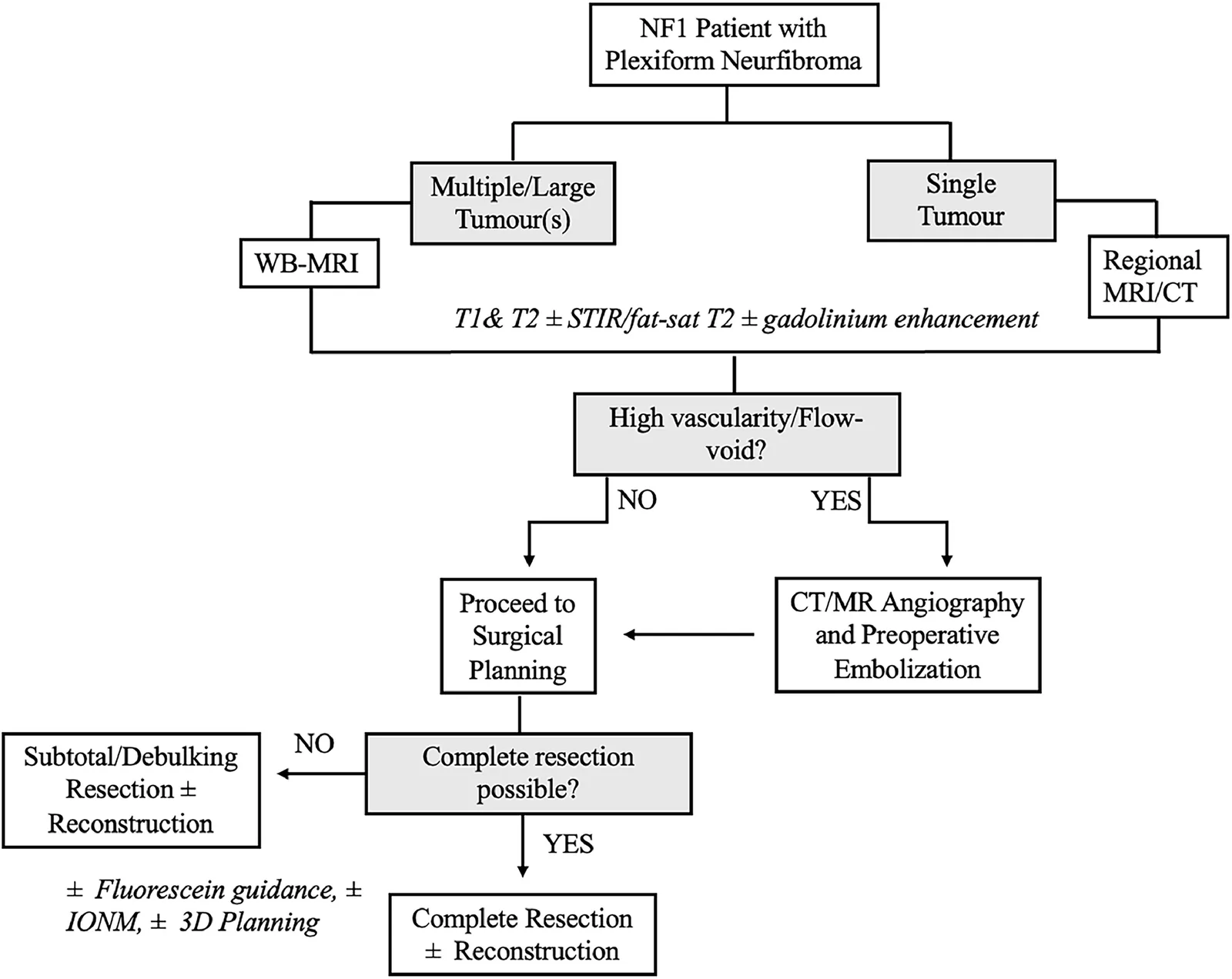
Proposed imaging-based perioperative workflow for the surgical management of plexiform neurofibromas. Start with WB-MRI or regional MRI/CT to define extent, perform CT/MR angiography ± targeted preoperative embolization for highly vascular lesions, then proceed to surgical planning with a decision between complete resection versus subtotal/debulking and reconstruction; consider intraoperative adjuncts (fluorescein, IONM, 3D planning) as available. NF1 = Neurofibromatosis type 1; WB-MRI = whole-body magnetic resonance imaging; CT = computed tomography; IONM = Intraoperative neurophysiological monitoring.

PNs of the limbs typically extend along the axis of peripheral nerves and infiltrate adjacent soft tissues, producing pain, deformity, and impairment of function. A common clinical presentation is a hyperpigmented, pendulous, multilayered soft tissue mass along the extremity. In a series of 17 patients (29 affected limbs) treated at a tertiary plastic surgery center, tumours confined to the subcutaneous plane or discrete nerve branches were amenable to subtotal or, in some cases, complete excision without sacrificing distal motor or sensory function.^55^ Meticulous microdissection permitted preservation of the principal nerve trunk while achieving durable local control, with no recurrences observed over three years of follow-up. In contrast, diffuse lesions that encase major neurovascular bundles often mandate compromise.^55^ Specifically, either debulking or deliberate nerve sacrifice may be needed to palliate symptoms when total resection is infeasible. One case report describes a 55-year-old woman with an 18- kg thigh PN that rendered ambulation impossible.^56^ Through a hybrid approach combining preoperative angiographic embolization and complete resection, the surgical team minimized intraoperative blood loss, resected the mass intact, and closed the defect primarily using redundant skin, ultimately restoring the patient’s ability to walk. Histopathology confirmed a benign, diffuse neurofibroma. For giant extremity PNs with extensive surface involvement, staged resections and advanced wound-care techniques, such as negative-pressure therapy, split-thickness skin grafting, and postoperative compression, have proven effective in managing lymphorrhea, facilitating closure, and optimizing cosmetic and functional outcomes, even in scenarios where amputation may otherwise be considered. Fig. 2 summarizes an imaging-based perioperative framework for PN surgery.

#### Special anatomical considerations

##### Paraspinal plexiform neurofibromas

Paraspinal PNs with epidural extension may cause radiculopathy or myelopathy and are managed more aggressively because decompression is often necessary to prevent permanent neurologic injury.^8^ Studies report favourable functional outcomes after resection of the intraspinal component, particularly when intervention is performed for progressive deficits.^7^^,^^8^ In one series of 13 patients with cervical cord compression (11 with multilevel involvement), subtotal resection of the intraspinal tumor led to resolution of weakness in 45% and halted further neurologic deterioration in 18%.^7^^,^^8^ In another series of 10 patients with progressive myelopathy or cauda equina dysfunction, gross-total resection of the intraspinal component was achieved in nine patients, with nine making complete neurological recoveries and the tenth showing marked improvement. Importantly, paraspinal PNs that compress the cord but produce no clinical signs do not automatically require intervention.

##### Symptomatic high proximal nerve lesions: brachial plexus and sciatic nerve

Symptomatic high proximal PNs involving the brachial plexus or sciatic nerve require multidisciplinary, individualized management because complete resection may cause major limb dysfunction and meaningful distal reinnervation may be limited by long regeneration distances. Management should first exclude MPNST or ANF using MRI, FDG PET-MRI or PET-CT when suspicious, and targeted biopsy when indicated.^23^^,^^57^ If ANF or MPNST is confirmed, primary resection should be pursued when safe and feasible.

For confirmed benign symptomatic PNs, treatment should prioritize preservation of existing neurologic function.^38^^,^^58^ The decision between surgery and medical therapy depends on tumor size, location, extent of nerve involvement, proximity to vital structures, and expected functional morbidity. When surgery is feasible, nerve-sparing enucleation is preferred for nodular lesions, with IONM used to help distinguish functional from nonfunctional.^59^, ^60^, ^61^ Subtotal resection or debulking is favored over radical resection when complete excision would compromise major nerve function. For unresectable or incompletely resectable benign PNs, MEK inhibitor may provide a function-preserving alternative to aggressive surgery^38^^,^^62^, ^63^, ^64^

When resection requires sacrifice of proximal nerve elements, conventional graft reconstruction may not restore meaningful distal function because delayed reinnervation can occur after irreversible motor endplate degeneration. In selected cases, nerve transfers may be considered because they shorten the distance to target muscles; however, expected recovery remains limited, particularly for proximal sciatic lesions or delayed presentations.^65^^,^^66^ Therefore, complete resection with parent nerve sacrifice should generally be reserved for confirmed or strongly suspected MPNST, severe progressive neurologic compromise, or life-/limb-threatening mass effect refractory to less morbid interventions.^23^^,^^67^

When MPNST is confirmed but complete negative-margin resection is not initially feasible, such as in proximal brachial plexus lesions extending toward the neuroforamina, management should be directed by a multidisciplinary sarcoma team.^68^ The goal is often to downstage disease and enable delayed resection using systemic therapy, radiotherapy, or combined modalities.^69^ Radiation planning is challenging near the spinal cord, brachial plexus, and neuroforamina, and highly conformal techniques such as proton therapy may be considered in select patients.^70^ If disease remains unresectable, treatment becomes primarily disease-controlling and may include definitive radiotherapy, additional systemic therapy, palliative surgery for symptom control, observation for stable asymptomatic disease, or best supportive care.

##### Orbitotemporal plexiform neurofibromas

These lesions are approached conservatively by consensus panels because of their complex anatomy and infiltrative behaviour. Debulking is reserved for progressive tumors that threaten vision or other critical functions.^7^^,^^8^

### Postoperative management & outcomes

#### Immediate postoperative care

Postoperative management following PN resection requires vigilant monitoring for hematoma, seroma, infection, wound dehiscence, and new or worsened neurological deficits.^30^^,^^71^ These risks are heightened in large, vascular tumors, where complications such as airway obstruction or spinal cord compression are more likely.^35^ Frequent neurological assessments are essential, as most deficits are mild and may improve over time.^71^ Closed-suction drains should be placed in large dead spaces and removed when output is <30 mL/24 h to reduce infection risk.^72^ ICU admission may be necessary for high-risk cases involving head and neck or spinal tumors, large tumor burden, significant comorbidities, or intraoperative instability.^73^ Negative-pressure wound therapy may be effective for both open wounds and high-risk closed incisions.^74^^,^^75^

#### Imaging and recurrence monitoring

Baseline postoperative MRI may be considered to assess for residual disease and establish a reference for future monitoring following partial or total resection of PNs.^76^ Long-term surveillance with annual MRI is appropriate for most patients with NF1, particularly those with large, growing, or incompletely resected tumors.^76^ High-risk patients, such as those with subtotal resection, deep or head and neck tumors, or younger age at surgery, may benefit from more frequent imaging, transitioning to annual scans if stable.^23^

#### Adjunctive therapies

MEK inhibitors, particularly selumetinib, are the standard of care for children aged ≥2 years with symptomatic, inoperable PNs, and are increasingly used in select adults with progressive or unresectable disease.^77^ Emerging data support the use of selumetinib in adults with inoperable PNs, demonstrating over 60% response rates, sustained tumor shrinkage, and improvements in pain and quality of life, similar to pediatric outcomes. These findings support its use in select adults with progressive or symptomatic disease.^78^ Monitoring for common adverse events (e.g., rash, diarrhea, fatigue, paronychia, elevated creatine kinase) remains important.^79^

#### Long-term outcomes

Neurological deficits may occur when tumors abut major nerves; most are transient but can be permanent, especially after complete resection.^6^ Tumor recurrence occurs in 18–68% of patients, more often in those with subtotal resection, younger age, or head and neck tumors.^80^ Persistent pain, functional impairment, and rare cases of malignant transformation highlight the need for long-term surveillance and supportive care.

## Conclusion

PNs present a formidable surgical challenge, demanding a deliberate integration of advanced imaging, targeted embolization, and individualized reconstruction to balance maximal tumor control with preservation of neurologic function and cosmesis. In this review, we have proposed an imaging-driven framework for staging and embolization selection and synthesized evidence on complete resection versus subtotal/debulking. We have also underscored the role of 3D planning and patient-specific guides in navigating complex anatomy, as well as the need for structured outcome reports on morbidity, recurrence, and quality-of-life measures. Ultimately, prospective, multicenter studies and multidisciplinary guidelines are needed to establish evidence-based pathways for surgical decision-making and optimize patient-centered outcomes.

## Funding

None.

## Ethical approval

Not required.

## Declaration of competing interest

None.
